# Mr. Brown, on Bleeding in Difficult Parturition

**Published:** 1809-03

**Authors:** B. Brown

**Affiliations:** Great Surry Street


### *To the Editors of the Medical and Physical Journal.*

Gentlemen,

MRS. G. aged 32, a powerful strong woman, of large stature, was taken in labour of her first child, on Sunday morning, at four o'clock. When I saw her, at twelve o'clock, it was reported to me, that her pains had been almost incessant, and as strong as she could possibly support. Accustomed to these expressions, I did not at first much regard them; but I found the representation had not been incorrect, for the distress induced by the pains, and their frequent and apparent violence were fully corroborated by my own observation. The os uteri I found slightly dilated, rigid, and unyielding, and its cervix appeared constricted internally, as if by a ligamentous band of some breadth. From this period till seven o'clock in the evening, in which time I saw her frequently, there did not appear any material alteration, excepting that the pains were more frequent, and the consequent distress to the patient greater, as the labour did not seem advanced by them. The os uteri, at this time, appeared dilated to nearly the size of half a crown, but equally rigid and thick as before, with the same degree of constriction at its cervix.

I now determined upon bleeding, as the most likely means of diminishing those powers of resistance which prevented the progress of the labour. I took away twenty ounces of blood. Its first effect appeared to protract the recurrence of the pains, and to render their remission more perfect in consequence. The subsequent effect upon the uterus, was to relax the constriction and diminish the rigidity of the os uteri; so that each succeeding pain produced its proper effect. The relaxation of the vagina and os externum were in equal ratio; and by ten minutes past twelve the child was born. Considering the time of

 bleeding bleeding and that at which the child was born, it was not reasonable to expect that the result could have been more speedy or favourable.

This being the first instance, in an extensive practice of thirteen years, in which I have resorted to bleeding under difficult parturition, its complete success induced me to believe that the publication of it might be useful to the younger practitioners of this island. If you are of the same opinion, you will find a place for it in your useful Repository.

I am, &c.

*Great Surry Street*, 19 *Feb.* 1809. B. BROWN.

